# Highly conjugated curcumin analog based copper complexes towards tuberculosis: synthesis, characterization and antimycobacterial activity

**DOI:** 10.1186/1471-2334-12-S1-P51

**Published:** 2012-05-04

**Authors:** J Joseph, G Boomadevi Janaki, K Nagashri, R Selwin Joseyphus, C Justin Dhanaraj

**Affiliations:** 1 Department of Chemistry, Noorul Islam Centre for Higher Education, Kumaracoil-629 180, Tamil Nadu, India; 2 Department of Chemistry, St John’s College, Anchal, Kerala, India; 3 Department of Chemistry, Anna University Tirunelveli, Tirunelveli, Tamil Nadu, India

## Background

Current first-line drugs for the treatment of tuberculosis consist of, i.e., isoniazid (INH), rifampin (RIF), ethambutol (EMB), pyrazinamide (PZA), and streptomycin (STR). Resistance to the first-line drugs causes treatment failure and necessitates the use of the drugs with a prolonged period of therapy. New anti-tuberculous agents, especially the ones with novel mechanisms of action are urgently required. Curcumin is a naturally occurring yellow pigment obtainable from the rhizomes of perennial herb Curcuma longa Linn., has been shown to act upon several important molecular targets in malignancy and inflammatory cascades and hence is used to treat various disorders including arthritis, Crohn’s disease, cardiovascular disorders, psoriasis, cancers, and other pathologies. However, poor water solubility and unsatisfactory pharmacokinetics of curcumin necessitate search for new curcumin analogs. In the present work, we described the synthesis and structural characterization of highly conjugated curcumin analogs (acetoacetanilide) Knoevenagel condensates, their Schiff bases, and corresponding copper conjugates.

## Method

The anti- *M. tuberculosis* activities of the compounds were determined using the MABA assay method.

## Results

The minimum inhibitory concentration of copper complexes has been performed against Mycobacterium tuberculosis strain H37Rv. It is observed that the MIC values of copper complexes (2-6 µg/ml) are slightly greater than the drug, ethambutol (1 µg/ml).

## Conclusion

Copper complexes have higher anti-mycobacterial activity than ligands due to the presence of highly conjugated curcumin analog system containing two azomethine groups and redox properties of metal. Based on our studies, we conclude that copper complex may be a promising candidate against tuberculosis.

